# Review of life expectancy in people with HIV in settings with optimal ART access: what we know and what we don't

**DOI:** 10.7448/IAS.15.6.18076

**Published:** 2012-11-11

**Authors:** C Sabin

**Affiliations:** University College London, London, UK

## Abstract

Life expectancy (LE) is an important indicator of health used widely by government and healthcare agencies to monitor trends over time and to determine resource allocation, as well as by insurance companies and pension providers. LE of the HIV-positive population has increased dramatically since the introduction of combination antiretroviral therapy (cART); indeed, it is now believed that LE may be similar to that of the general population in some subgroups. There are, however, specific subgroups in which LE remains substantially impaired. The impact of HIV and of cART on mortality may be expressed in several ways. LE itself provides an estimate of the average additional number of years that an individual would be expected to live beyond a particular age. However, the detrimental impact of HIV may also be described in terms of the number of years of life lost or the gains in LE if HIV were to be eliminated as a cause of morbidity in the population. My presentation will start with a description of the different methods that researchers have used to describe the mortality outcomes of those with HIV, and the impact of cART on these. I will then consider how LE in the HIV-positive population has changed over time and will describe the impact of demographic factors (e.g. gender, age, ethnic group) on LE. To investigate the circumstances under which LE may return to normal levels, I will also consider the potential impact of timely diagnosis and linkage into care, continued engagement with care, optimal initiation of cART and maintenance of viral suppression on LE. Finally, I will discuss some of the limitations of the approaches used to estimate LE, with particular emphasis on the confounding effects of lifestyle and behavioural factors when making any comparison with LE in the general population.

